# Use of Limestone Sludge in the Preparation of ɩ-Carrageenan/Alginate-Based Films

**DOI:** 10.3390/ma17071668

**Published:** 2024-04-05

**Authors:** Pedro Adão, Maria da Luz Calado, Wilson Fernandes, Luís G. Alves, Leonor Côrte-Real, Mafalda Guedes, Ricardo Baptista, Raul Bernardino, Maria M. Gil, Maria Jorge Campos, Susana Bernardino

**Affiliations:** 1 MARE-ARNET and Escola de Turismo e Tecnologias do Mar, Instituto Politécnico de Leiria, 2520-614 Peniche, Portugalraul.bernardino@ipleiria.pt (R.B.); maria.m.gil@ipleiria.pt (M.M.G.); mcampos@ipleiria.pt (M.J.C.); 2 Centro de Química Estrutural, Institute of Molecular Sciences, Department of Chemical Engineering, Instituto Superior Técnico, Universidade de Lisboa, Av. Rovisco Pais 1, 1049-001 Lisboa, Portugalleonor.corte-real@tecnico.ulisboa.pt (L.C.-R.); 3 UnIRE, ISEL, Instituto Politécnico de Lisboa, Av. Conselheiro Emídio Navarro 1, 1959-007 Lisboa, Portugal; 4 LaPMET-CeFEMA, Instituto Superior Técnico, Universidade de Lisboa, Av. Rovisco Pais, 1049-001 Lisboa, Portugal; 5 LAETA, IDMEC, Instituto Superior Técnico, Universidade de Lisboa, Av. Rovisco Pais, 1049-001 Lisboa, Portugal; 6 Laboratory of Separation and Reaction Engineering-Laboratory of Catalysis and Materials (LSRE-LCM), School of Technology and Management (ESTM), Polytechnic Institute of Leiria, 2520-614 Peniche, Portugal; 7 ALiCE—Associate Laboratory in Chemical Engineering, Faculty of Engineering, University of Porto, Rua Dr. Roberto Frias, 4200-465 Porto, Portugal

**Keywords:** limestone sludge, ɩ-carrageenan, sodium alginate, algal polysaccharides, polysaccharide films

## Abstract

The use of processed limestone sludge as a crosslinking agent for films based on Na–alginate and ɩ-carrageenan/Na-alginate blends was studied. Sorbitol was tested as a plasticizer. The produced gel formulations included alginate/sorbitol and carrageenan/alginate/sorbitol mixtures, with tested sorbitol concentrations of 0.0, 0.5 and 1.0 wt%. The limestone sludge waste obtained from the processing of quarried limestone was converted into an aqueous solution of Ca^2+^ by dissolution with mineral acid. This solution was then diluted in water and used to induce gel crosslinking. The necessity of using sorbitol as a component of the crosslinking solution was also assessed. The resulting films were characterized regarding their dimensional stability, microstructure, chemical structure, mechanical performance and antifungal properties. Alginate/sorbitol films displayed poor dimensional stability and were deemed not viable. Carrageenan/alginate/sorbitol films exhibited higher dimensional stability and smooth and flat surfaces, especially in compositions with 0.5 wt% sorbitol. However, an increasing amount of plasticizer appears to result in severe surface cracking, the development of a segregation phenomenon affecting carrageenan and an overall decrease in films’ mechanical resistance. Although further studies regarding film composition—including plasticizer fraction, film optimal thickness and film/mold material interaction—are mandatory, the attained results show the potential of the reported ɩ-carrageenan/alginate/sorbitol films to be used towards the development of viable films derived from algal polysaccharides.

## 1. Introduction

Limestone residue waste (limestone sludge) is a by-product of the ornamental stone quarry and processing industries [1,2]. A minimum amount of 9–10% of wet sludge is typically generated [3,4] and usually landfilled at sludge ponds [1,2]. Dimension limestone is considered one of the most popular stones in the global market because of its lower cost and superior ability to be polished similar to marble. According to a report by the United States Geological Survey, dimension limestone constitutes 45% of the USA’s market [5]. Given the large volume produced worldwide and its persistence, these residues present an environmental concern regarding the impact on local air, land and water quality [4]. Nevertheless, limestone waste may be recycled and find use as a raw material for other applications, depending on its composition. For instance, marble stone sludge may find use as a potential component in construction materials by incorporation into mortar, bricks and cement [6,7,8,9]. The average composition of limestone sludges includes more than 95 wt% calcium carbonate (CaCO_3_) [2], making it highly desirable for a range of applications, including agricultural liming, acid neutralization, flux for iron sinters, cement manufacturing, soil stabilization, glass manufacturing and gas fuel desulfurization [1,3]. Another potential use arose more recently, considering that Ca^2+^ is often used in the crosslinking of polysaccharide films and coatings. That is, the limestone sludge can be used as a source of calcium ions in the production of bioplastic films and coatings with improved mechanical properties [10]. Two such polymers, of particular interest due to their ability to form water-insoluble gels in the presence of calcium ions, are sodium alginate and ɩ-carrageenan [11,12]. Both have a strong affinity for Ca^2+^ and can form gels that are able to be arranged into film sheets when the solvent is removed [13], showing high potential for replacement of conventional petroleum-based packaging films [11,12]. In addition, being obtained from potentially renewable algal biomass, alginates and carrageenans may contribute to reducing the demand for fossil-based feedstocks for the production of petroleum-based plastic films [10,14].

Alginate is a polyanionic salt of alginic acid, typically extracted from brown marine macroalgae from species of the Macrocystis, Laminaria and Ascophyllum taxonomic genera [15]. Structurally, sodium alginate is a linear heteropolymer consisting of two uronic acids: D-mannuronic acid (M) and L-guluronic acid (G), joined by a -1,4-glycosidic linkage [16]. The polymer chain is divided into alternating segments of M-blocks, G-blocks and MG-blocks, with no ordered sequence [16].

Depending on the medium pH, ions such as Ca^2+^ can be chelated by carboxylate groups on the G-blocks, participating in intermolecular crosslinking and leading to the immobilization of alginates [15,16,17]. This is because the G residues fold and stack under the bond interaction, which causes the structural transformation of adjacent alginate chains from random coils into an ordered ribbon-like structure. The resulting chain entanglement renders a hydrogel with a three-dimensional net structure [17]. Important properties of alginate polysaccharides are their biocompatible, biodegradable and hydrophilic behavior; the ability to retain water (>98%); and the gelling, thickening, and stabilizing properties [15].

On their turn, carrageenans are anionic sulfated linear polysaccharides of D-galactose and 3,6-anhydro-D-galactose extracted from the marine red macroalgae *Chondrus crispus*, *Kappaphycus alvarezii* and *Eucheuma denticulatum* [10,18]. They mainly consist of alternating 3-linked β-D-galactopyranose (G-units) and 4-linked α-D-galactopyranose (D-units) or 4-linked 3,6-anhydro-α-D-galactopyranose (DA-units) that form the disaccharide repeating unit of carrageenans [18]. The number and position of the ester sulfate groups categorize carrageenans into three primary classes—k-, ɩ- and l [17,18]. Ι-carrageenan carries two sulfate groups per disaccharide unit, correspondent to a calculated sulfate content of 33 wt% [10,18].

The gelation of carrageenans involves two separate and successive steps: coil-to-helix conformational transition, followed by helix aggregation and subsequently their cation-dependent aggregation [17,18]. The presence of a suitable cation able to intrude between double helices and facilitate their approach by neutralizing the charges of sulfate groups, is an absolute requirement for gelation [18]. All alkali metal ions can induce gelation, but regarding ɩ-carrageenan, Ca^2+^ is considerably more effective than other ions [10,11]. Gelation results from the formation of a three-dimensional network of double helices, resulting from the crosslinking of adjacent spiral chains, with sulfate groups oriented towards the exterior [18]. Ca^2+^-containing carrageenan bioplastics produce clear, elastic and soft gels, able to form compact films.

When blended, Na-alginate and ɩ-carrageenan are able to establish a high number of hydrogen bonds since they hold a high content of carboxyl groups and hydroxyl groups, respectively. As a result, carrageenan/alginate gels consist of a crosslinked double network linked through hydrogen bonding and ionic interaction [19]. Such a network maintains a relatively stable form and makes the sodium alginate and carrageenan intermolecular spacing decrease so that the spatial network structure becomes increasingly compact in gelation [19].

As raw materials for bioplastics, both alginate and carrageenan are individually easily soluble and have poor mechanical properties (brittle and stiff) [13]. Also, they both have water-soluble properties, low intermolecular distances and low polymer chain mobility, requiring the addition of a plasticizer to improve physical and mechanical properties [13]. The addition of a plasticizer serves to mechanically reduce the intermolecular interactions between polymer chains, increasing chain mobility, so that the bioplastic becomes more elastic [13]. In this work, sorbitol was selected as a plasticizer since the gelation process of both sodium alginate and carrageenan is strongly affected by sorbitol [20]. Also, sorbitol is non-toxic, while increasing sorbitol concentration accelerates the process of bioplastic degradation [21]. This way, films remain environmentally friendly products when sorbitol is used for processing additive degradation [21].

Given the affinity of both alginate and ɩ-carrageenan towards Ca^2+^, the conversion of a calcium-rich limestone sludge into a suitable liquid crosslinking reagent is expected to render crosslinked water-resistant alginate and/or ɩ-carrageenan-derived films. This is expected to be an important contribution to the development of bio-based films aimed to replace conventional petroleum-based packaging films, thus toiling to reduce the demand for fossil-based feedstocks [10,11]. Additionally, this solution promotes the valorization of limestone sludge waste, targeting the valorization of mineral and marine resources towards circular economy goals.

In this context, this work aims to (i) obtain a viable method for the conversion of limestone sludge residue into a crosslinking agent suitable for films based on sodium alginate and/or ɩ-carrageenan and (ii) develop film formulations that are chemically and dimensionally stable and display adequate mechanical properties.

## 2. Materials and Methods

### 2.1. Materials

MARFILPE (Batalha, Portugal) graciously provided the sludge, which resulted from the processing of Vidraço de Moleanos limestone quarried in the region of Alcobaça, Portugal). Vidraço de Moleanos is a whitish-brown limestone (apparent density 2.570 g/cm^3^, max. 0.75% impurities), coarsely calciclastic, slightly oolitic and abundantly bioclastic. Commercial sodium alginate (food grade, Farmaquímica Sur S.L., Malaga, Spain) and ɩ-carrageenan (food grade, Sosa, Barcelona, Spain) in powder form was the algal polysaccharide used throughout. D-(-)sorbitol (VWR, purity > 97%, 1.524 g/cm^3^, Barcelona, Spain), also in powder form, and was used as a plasticizer.

### 2.2. Production and Characterization of Crosslinking Solution from Limestone Sludge

The limestone sludge was dried at room temperature until a compact solid material was obtained. Its calcium content was determined using Inductively Coupled Plasma Optical Emission Spectroscopy (Section 2.5.1). The material was deagglomerated into a loose powder and used to prepare a suspension (600 mL) with 33 wt% solids. Concentrated hydrochloric acid (VWR, purity > 36.2%, 1.18 g/cm^3^, Barcelona, Spain) was added dropwise while stirring with a magnetic stirrer (ARE heating magnetic stirrer, VELP Scientifica, Usmate, Italy) until effervescence subsided. The addition of the acid was very slow to control spilling and excessive foaming. The suspension was stirred with a magnetic stirrer for 10 min and adjusted to pH 7 with an aqueous NaOH 4 M solution (LabChem, Edenvale, South Africa). Vacuum filtration with a vacuum pump (Vacuubrand ME 2C, Wertheim, Germany) was then carried out, using quantitative hardened filter paper (ref. 2240, Filter-lab, Barcelona, Spain). The filtrate was stirred at room temperature for 24 h, after which the liquid was again vacuum filtered. The new filtrate was transferred to a volumetric flask and distilled water was added until 1 L of liquid was obtained. The crosslinking agent used for the gel formulations without sorbitol consisted of this solution diluted with distilled water in a 1:1 ratio. A similar crosslinking agent containing sorbitol (1 wt%) was also prepared and used as a crosslinking agent for formulations containing sorbitol. The corresponding formulation labels are marked with * (please refer to Table 1).

The concentration of Ca^2+^ equivalents in the crosslinking agent was estimated by complexometric titration [22]. A solution of ethylenediaminetetraacetic acid disodium salt dihydrate, EDTA, 0.0101 M (Na_2_H_2_EDTA.2H_2_O, Biochem, purity > 98%, Karlsruhe, Germany) was used as the titrant. The analyte was prepared by dissolving 1 g of NaOH in 200 mL of distilled water and then adding 0.5 mL of the crosslinking agent solution. A cloudy mixture formed, which was topped with distilled water to reach 250 mL and then homogenized. After 5 min rest, 10 mg of calconcarboxylic acid indicator (Fluka, max water 7%, 1.6 g/cm^3^, Madrid, Spain) was added. The solution was stirred until the full dissolution of the indicator, with the obtention of an apparently clear red sample stock. Six aliquots with 25 mL of the liquid crosslinking agent were titrated with the EDTA solution until a clear blue color was obtained.

### 2.3. Preparation of Alginate Films

The alginate films were prepared based on a procedure proposed by other authors [23,24,25]. An aqueous casting solution of 1% alginate (ALG) was prepared by dissolution of solid sodium alginate in distilled water at room temperature using a magnetic stirrer. After the dissolution of the alginate, solid SORB was directly added to the ALG solution so that an alginate/sorbitol weight ratio of 1:1 was achieved. After the dissolution of SORB, the resulting viscous solution was vacuum filtered using a vacuum pump (Vacuubrand ME 2C, Wertheim, Germany) at least three times through a four-layer synthetic cloth to remove insoluble contaminants and sonicated in an ultrasonic bath (USC600-TH Ultrasonic Cleaner, VWR, Barcelona, Spain) to remove trapped air. This solution was then used to prepare ALG/SORB films (Table 1) by mold casting; polystyrene Petri dishes (140 mm diameter) were used as molds. An amount of 100 mL of filmogenic solution was poured into each mold and stored in an oven (IF110, Memmert, Schwabach, Germany) at 40 °C (50% vent, 10% flap) for 24 h. Some of the dried precursor films were sprayed with 1 wt% sorbitol aqueous solution until the entire upper surface was wet; the others remained dehydrated. All dishes were covered with their respective lid and allowed to rest at room temperature for 24 h, after which 40 mL of the crosslinking agent (Section 2.2, a 1:1 mixture of dissolved sludge and distilled water) was poured upon. D-(-)sorbitol (1%) was included in the crosslinking agent used with the ALG/SORB formulations. The films were carefully peeled from the mold while immersed, and the other side was also allowed to contact the solution by gently stirring the mold and rotating the loosened film (1 min total time immersed). The film and the mold were then washed with distilled water and the film was repositioned in the mold, covered and allowed to dry at room temperature.

### 2.4. Preparation of ɩ-Carrageenan/Alginate Films

The procedure for preparation of ɩ-carrageenan/Na-alginate films was similar to that used for the ALG/SORB films, except for the hydration step previous to crosslinking, which was not carried out. A solution of ɩ-carrageenan/alginate (1 wt% total polysaccharide) was prepared by dissolving 0.75 wt% of solid ɩ-carrageenan and 0.25 wt% of solid sodium alginate in an appropriate volume of distilled water while vigorously stirring with a magnetic stirrer at room temperature (75-CRG/25-ALG films, Table 1). In addition, formulations with D-(-)sorbitol were prepared by adding 0.5 wt% or 1 wt% of the polyol to the polysaccharide solutions (CRG/ALG/SORB films, Table 1).

The resulting viscous solution was vacuum filtered with a vacuum pump (Vacuubrand ME 2C, Wertheim, Germany) through a four-layer synthetic cloth and sonicated in an ultrasonic bath (USC600-TH Ultrasonic Cleaner, VWR, Spain). An amount of 74 mL of filtered solution was then poured into 110 mm polystyrene Petri dishes, which were stored in an oven at 40 °C (50% vent, 10% flap) for 24 h. After that period, 20 mL of the crosslinking agent (Section 2.2, a 1:1 mixture of dissolved sludge solution/distilled water) was poured upon the dried precursor films. D-(-)sorbitol (1 wt%) was also included in the crosslinking solutions for the films containing the polyol. The film was carefully peeled from the mold while immersed and both sides were allowed to contact the solution by gently stirring the mold and rotating the film (2 min total time immersed). The film and the mold were washed with distilled water and the film was again placed on the mold, covered and allowed to dry under room conditions. Samples were stored for 1 month before characterization.

### 2.5. Chemical Characterization by Spectroscopy Techniques

The sludge was characterized regarding calcium concentration using Inductively Coupled Plasma Optical Emission Spectroscopy (ICP-OES). The produced films were characterized regarding chemical and microstructural features using spectroscopy and microscopy techniques, respectively. Measurement, analysis and observation were always carried out on the films’ surface, which dried freely.

#### 2.5.1. Inductively Coupled Plasma Optical Emission Spectroscopy

ICP-OES analysis was carried out (iCAP 7000, ThermoFisher Scientific, Waltham, MA, USA) to quantify available calcium in the limestone sludge. Nitric acid (PanReac, purity > 65%, 1.39 g/cm^3^, Barcelona, Spain) and hydrochloric acid 37% (VWR, purity > 36.2%, 1.18 g/cm^3^, Barcelona, Spain) were mixed in 1:3 molar ratio to prepare aqua regia, which was used to digest the sludge samples. Analysis took place under argon using the following plasma conditions: camera temperature, 45 °C; optics temperature, 38 °C; pump rate, 50 rpm; auxiliary gas flow, 0.5 L/min; nebulizing gas flow, 0.7 L/min. Other specific elements investigated (Al, P, S, Fe) were quantified by comparison with a calibration curve, built using calibration standards with known concentrations of those elements.

#### 2.5.2. Nuclear Magnetic Resonance

The composition and purity of the produced materials were controlled via ^1^H Nuclear Magnetic Resonance (Bruker 400 MHz Avance II NMR spectrometer, 5 mm BBO probe, Bruker BioSpin AG, Fällanden, Switzerland). Spectra processing was carried out with ssNake (v1.3) program [26]. ^1^H chemical shifts (δ) are expressed in parts per million (ppm) referenced to sodium trimethylsilylpropanesulfonate (DSS). The ^1^H NMR spectra were obtained at 60 °C, with 128 scans and solvent suppression [27]. D_2_O, DMSO-d_6_ and sodium trimethylsilylpropanesulfonate (all from Eurisotop, Saint-Aubin, France) were used as received.

#### 2.5.3. Attenuated Total Reflectance Fourier Transform Infrared Spectroscopy

The chemical structure of the films and interaction between polysaccharides and their functional groups were assessed via Fourier Transform Infrared Spectroscopy-Attenuated Total Reflectance (FTIR-ATR). Acquisition was carried out using a Spectrum Two spectrometer (PerkinElmer, Shelton, Connecticut, US) equipped with a UATR Two (Perkin Elmer, Shelton, CT, USA) universal ATM module (diamond cell). Acquisition took place at room temperature, registering four scans in the 4000–500 cm^−1^ range, with 4 cm^−1^ resolution.

### 2.6. Antifungal Assays

The antifungal activity of the produced 75-CRG/25-ALG biofilm was tested against the pathogenic strain of *Aspergillus fumigatus* (DSMZ 819) obtained from the German Collection of Microorganisms and Cell Cultures (Leibniz, Germany) by using the agar disk-diffusion method. This filamentous fungus was chosen because it represents a ubiquitous airborne species distributed indoors and outdoors, which might occur in several food items and cause aspergillosis in immunosuppressed individuals [28]. Moreover, this species belongs to a genus that has been used as a model for many types of studies [29].

Firstly, *A. fumigatus* was grown on Potato Dextrose Agar (PDA) medium for one week at 28 °C to induce the production of spores. A portion of spores was carefully scraped from the mycelium surface with a sterile blade and suspended in a 0.1% aqueous solution of Tween 20. The number of spores in the solution was determined, and the concentration of spores was adjusted to 1 × 10^4^ spore/mL [30]. Then, 100 µL of the spore solution was inoculated and uniformly spread onto PDA plates.

The film was cut into several 1.5 cm circular disks, which were sterilized in a laminar flow cabinet (Telstar Bio II Advance Plus, Barcelona, Spain) under UV radiation for 30 min on each side [31] and aseptically transferred to three inoculated plates (two or three disks per plate). The plates were then incubated for 7 days at 28 °C and monitored daily for the presence of inhibition halos. Additional inoculated PDA plates without film disks were used as controls.

### 2.7. Morphologic Characterization

Films were first examined macroscopically. Afterward, scanning electron microscopy (SEM) (S2400 Hitachi, Tokyo, Japan) was used to observe the films’ surface and characterize their microstructure, including the presence of defects (pores, cracks, inclusions). Elemental microanalysis was carried out with energy dispersive spectroscopy (EDS) (Oxford Instruments Inca pentaFETx3, High Wycomb, UK) coupled to the microscope. Samples were previously coated with an Au–Pd alloy to avoid an accumulation of electrical charges during observation. Quantitative micrographic analysis was carried out using a minimum of five images of each sample at a magnification of at least 2000×.

### 2.8. Characterization of Mechanical Performance

Tensile tests were carried out on a universal electromechanical testing machine (INSTRON, 5544, Norwood, US) equipped with a 100 N load cell (INSTRON, Norwood, US) and a video strain gauge (INSTRON, SVE I, Norwood, MA, USA). Tests were carried out until sample fracture, with a speed of 1 mm/min. Both the Young’s modulus and the ultimate tensile stress (UTS) of the materials were calculated according to the ASTM D638 standard test method for the tensile properties of plastics. At least three (*n* = 3…6) samples with 15 mm width and 55 mm gauge length (over 95 mm long) were used per film composition. Film thickness was measured using a micrometer (MAUSER, Baden-Württemberg, Germany); four samples of each composition were tested for reproducibility assessment.

## 3. Results and Discussion

### 3.1. Composition of the Stone Sludge and of Crosslinking Solution

ICP-OES analysis of the starting sludge material indicated a calcium equivalent content of 425.60 ± 28.61 mg/g in addition to other selected elements, such as Al (0.0877 ± 0.0027 mg/g), P (0.0192 ± 0.0006 mg/g), S (1.0928 ± 0.0588 mg/g) and Fe (0.1865 ± 0.0306 mg/g). The existence of Cd and Hg was also analyzed, and contents below 0.00004 mg/g for Cd and 0.0014 mg/g for Hg were obtained. The concentration of equivalent calcium ion in the prepared crosslinking solution determined complexometric titration was 1.79 ± 0.02 M.

### 3.2. Characterization of the Produced Crosslinked Films

#### 3.2.1. Alginate Films

Preliminary experiments with alginate-only films confirmed the typically stiff and brittle mechanical behavior, which is a major issue in processing functional alginate films [13]. This behavior is expected to result from the considerable extent and rate of the reactions taking place during crosslinking. On the one hand, the driving force for shrinkage mainly comes from the energy gain enabled by the decrease of the gel interfacial area [32], which is a powerful incentive for gel densification [32]. This may originate syneresis, i.e., spontaneous liquid expulsion promoted by gel shrinkage and densification, without solvent evaporation [32,33]. On the other hand, the significant reduction in the interfacial area corresponds to a high crosslinking reaction rate [32]. This leads to the formation of a heterogeneous three-dimensional gel structure due to very fast and irreversible Ca^2+^ ion binding to the alginate polymer chains (Equation (1)), leading to syneresis [15,33,34]. As the gel shrinks, the polymer clusters pack together more closely and chain mobility decreases and consequently stiffens the structure [32]. Additionally, shrinkage is bound to bring together the reactive hydroxyl groups of the alginate chains, and new chemical bonds form that further contribute to the rigidity of the gel structure [32]. This results in very brittle alginate films, making handling and further processing very difficult.


(1) $${\mathrm{Na}}_{n}{\mathrm{alginate}}_{\left(\mathrm{solution}\right)}+\frac{n}{2}{\mathrm{Ca}}_{\left(\mathrm{solution}\right)}^{2+}\;\to {\mathrm{nNa}}_{\left(\mathrm{solution}\right)}^{+}+{\;\mathrm{Ca}}_{n/2}{\mathrm{alginate}}_{\left(\mathrm{gel}\right)}$$

**Figure 1:**
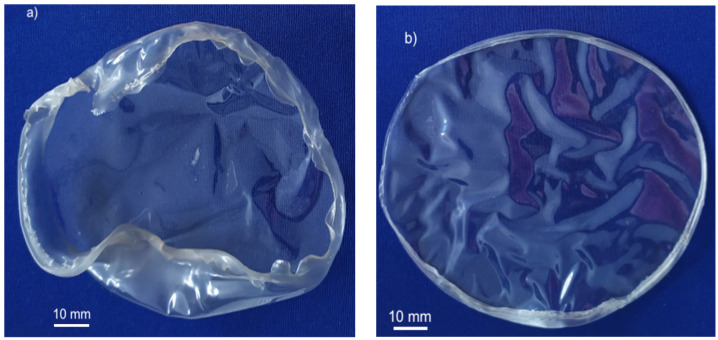
Crosslinked alginate/sorbitol films (100-ALG/100-SORB*): (**a**) without hydration step; (**b**) with hydration step, using 1% sorbitol aqueous solution.

In this current work, a sorbitol plasticizer was used to address these issues in the expectation of modifying the viscoelastic properties of the gel, and alginate/sorbitol films (100-ALG/100-SORB*) were first prepared. Yet, without hydration before crosslinking (Section 2.5), the films deformed and cracked during solvent removal (Figure 1a). Thus, another route to increase the films’ flexibility was superimposed: the addition of a plasticizer to the crosslinking solution (external plasticization). External plasticization corresponds to the addition of a compound that is not chemically bonded to the polymer chain, and it can therefore be lost by evaporation, migration or extraction [16,35]. D-(-)-sorbitol was used for this purpose in the form of a 1 wt% sorbitol aqueous solution used to spray the upper surface of the dried precursor film before crosslinking (Section 2.3). Interaction with the polymer requires sorbitol to penetrate the alginate gel and interpose itself between the polymer chains [36], toiling by means of its solvent or swelling power [35]. The first mechanism seems to predominate when sorbitol content is low, while the second predominates for high sorbitol concentration [21]. It should be mentioned that the specific concentration depends on the chemical and physical structure of the macromolecules, i.e., on the intermolecular forces to overcome [21]. Nevertheless, although more flexible, these films still underwent visible shrinkage and deformation upon crosslinking and drying (Figure 1b), displaying linear retraction between 21 and 32% and significant waviness and warping (Figure 1a).

Although films that underwent the spray hydration step (Figure 1) were significantly improved compared to films prepared without the spray hydration, the expected outcome of developing a satisfactory processing system was not achieved. This is expected to result from the excessive fraction of sorbitol since the associated stabilization mechanism is steric hindrance [16,37,38]. The flexible and (comparatively) low-molecular-weight sorbitol chains infiltrate the gel and extend into the surrounding liquid phase, creating a physical barrier that prevents the polymer chains from coming into close contact [35]. Aggregation is inhibited, decreasing gel viscosity and increasing chain mobility and solution stability, thus enabling flexibility over time [16,34]. Yet, when the dispersant exceeds some critical concentration value [34], sorbitol molecules may interact to create a bulky gel-like structure within the system, which results in dimensionally unstable and brittle films [16]. 

#### 3.2.2. Films of ɩ-Carrageenan/Alginate

**Figure 2:**
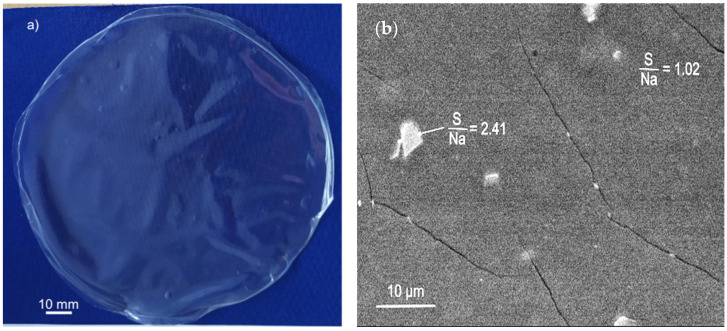
Crosslinked films with 75-CRG/25-ALG formulation: (**a**) macrography, showing warping and surface waviness; (**b**) low magnification SEM image, showing insufficient miscibility between the two polysaccharides. (Films and crosslinking solution without sorbitol).

In the face of the described issues displayed by alginate/sorbitol films, formulations based on ɩ-carrageenan/alginate mixtures, reportedly more stable than the individual polymers [19], were developed. The polysaccharide mixture is expected to form a 3D gel double network, attenuating the drawbacks of low mechanical strength, low dimensional stability and the tendency of syneresis of the individual gels [19,39]. The 75-CRG/25-ALG formulation (Table 1) was the first tested and, even in the absence of a sorbitol plasticizer, the films appeared to be more flexible and less prone to deformation during crosslinking and drying than alginate films (Figure 2a). Yet, they were still difficult to handle without breaking, and after 1 month, their measured linear retraction was 14.2 ± 4.9% (Table 2). Several issues are expected to contribute to the apparently low film performance. On the other hand, the high dimensional retraction suggests that significant loss of water by syneresis and slow evaporation took place after the production of the film (this composition rendered the films with the lowest measured thickness, around 45 µm, Table 2). On the other hand, while film production took place at room temperature, the glass transition temperature is 115 °C for Na-alginate [40] and 99 °C for ɩ-carrageenan [41]. Thus, although the two components are soluble in water and miscible in each other, their mobility is low. The achieved physical blend is insufficient to result in full miscibility, and the overall film structure at the microscale remains heterogeneous [40]. This is in good agreement with SEM observation (Figure 2b), showing irregular particles dispersed in a more homogeneous background. Results of the EDS elemental identified a S/Na molar ratio of around 1.0 in the background and 2.4 in the particles, indicating that the particles contain a higher amount of ɩ-carrageenan than the background.

**Figure 3:**
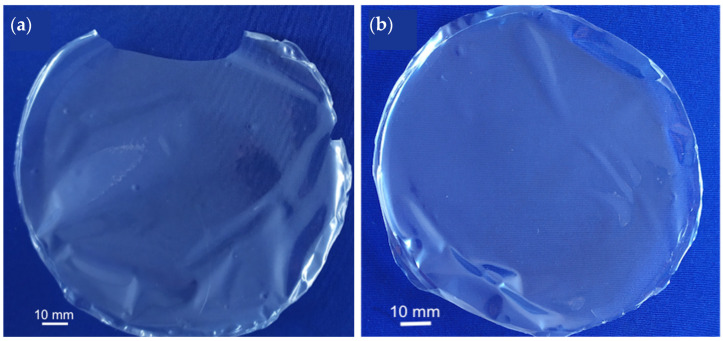
Crosslinked CRG/ALG films with increasing amount of D-(-)sorbitol: (**a**) 75-CRG/25-ALG/50-SORB and (**b**) 75-CRG/25-ALG/100-SORB (crosslinking solution without sorbitol in both films).

The addition of a plasticizer (50% and 100%, based on the total mass of algal polysaccharides) introduced important changes. The corresponding crosslinked films, 75-CRG/25-ALG/50-SORB (Figure 3a) and 75-CRG/25-ALG/100-SORB (Figure 3b), proved more resistant to handling than alginate/carrageenan films. This is expected to result from the presence of plasticizer: sorbitol’s small molecules distribute among the larger polysaccharides’ network, causing the chains to pack less densely and reducing the van der Waals forces binding the polymers together [34]. This reduces intermolecular interactions between alginate chains, reduces viscosity, allows for chain mobility (increasing film flexibility), and retains the gel’s ability to resist syneresis during densification [34,37]. Also, D-(-)sorbitol’s glass transition takes place at −1.3 °C [42]; thus, the polymer is fluid at room temperature and toils to reduce the overall temperature of the glass transition of the system, decreasing viscosity [34]. Also, the fact that sorbitol is hygroscopic brings more water to the system [42]. All of these factors enhance sorbitol’s plasticizer ability [34]. The addition of sorbitol also increases film thickness expectedly because bringing another species to the system increases the gel’s solid content, resulting in increased thickness [13,43]. This is in good agreement with the thickness increase with sorbitol concentration increase, corresponding approximately to 45 µm, 51 µm and 52 µm for 0 wt%, 0.5 wt% and 1 wt% sorbitol, respectively (Table 2).

**Figure 4:**
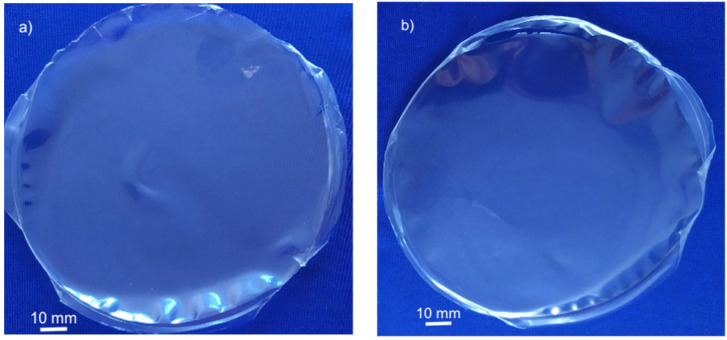
Crosslinked CRG/ALG films with increasing amount of D-(-)sorbitol: (**a**) 75-CRG/25-ALG/50-SORB* and (**b**) 75-CRG/25-ALG/100-SORB* (crosslinking solution containing sorbitol in both films).

Films’ flexibility and handling ability appears to increase even further when sorbitol is also present in the aqueous crosslinking solution (1 wt%). The 75-CRG/25-ALG/50-SORB* (Figure 4a) and 75-CRG/25-ALG/100-SORB* (Figure 4b) films still present an irregular rim, which is possibly a size effect resulting from the small mold area [13,37]; film waviness notably decreased.

**Figure 5:**
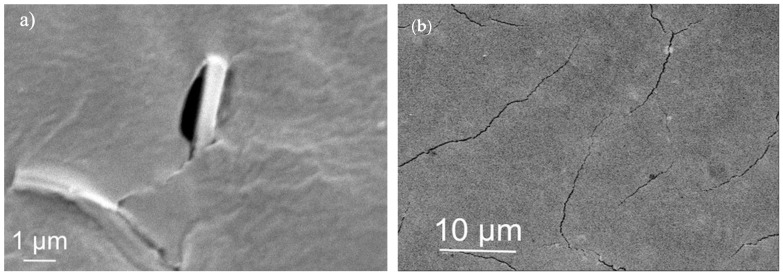
Examples of defects found in the films: (**a**) micrometric carrageenan-rich self-inclusions disrupting film uniformity (75-CRG/25-ALG/50-SORB film); (**b**) micrometric pores and cracks (75-CRG/25-ALG/100-SORB film).

All formulations maintained a smooth, clear and transparent appearance after 1 month. Yet, despite the macroscopic uniformity of the films, important defects arose at the microscale, including inclusions (Figure 5a), fractures and pores (Figure 5b).

**Figure 6:**
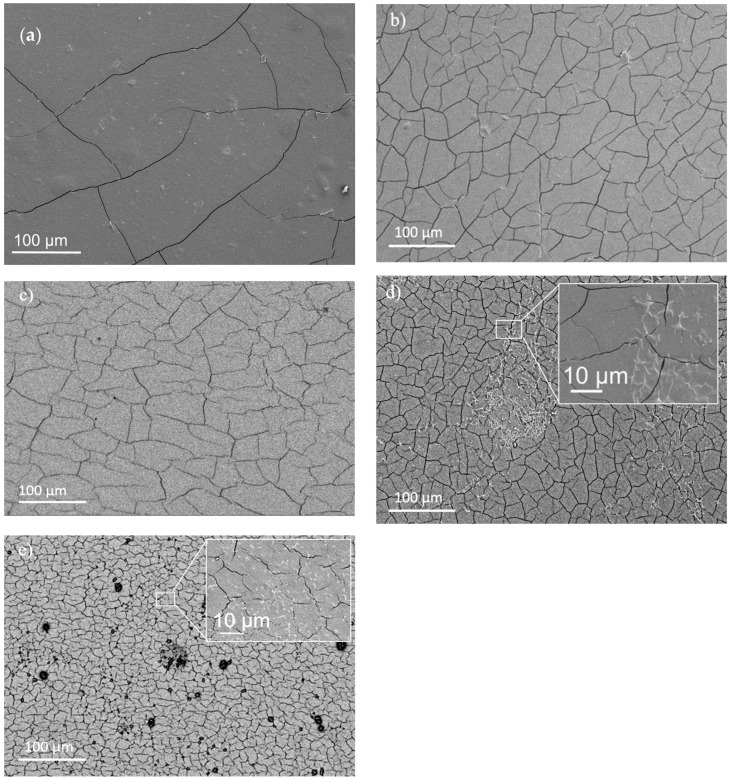
Low magnification images showing an overall view of films’ surface.(**a**) 75-CRG/25-ALG, (**b**) 75-CRG/25-ALG/50-SORB, (**c**) 75-CRG/25-ALG/100-SORB, (**d**) 75-CRG/25-ALG/50-SORB*, and (**e**) 75-CRG/25-ALG/50-SORB* (sorbitol present in the crosslinking solution). Superimposed micrographs show a higher magnification image of the corresponding film.

The most striking microscopical feature in all the produced films is the mud-cracking fracture pattern (Figure 6), which is expected to result from volume changes imposed during solvent removal. In fact, all drying processes are accompanied by significant gel shrinkage and densification, introducing stresses that deeply affect the final film microstructure [32]. Strain develops because the top layer tightens while the material below stays the same size, wherein film tightening imposes compressive stress on the substrate and tensile stress on the film. If the gel adheres strongly to the mold surface, there will be essentially no strain at the gel/mold interface, and the drying gel will contract only in the perpendicular direction. When this strain becomes large enough, cracks form in the dried-up surface to relieve the strain [44]. Individual cracks spread and joined up, forming a polygonal, interconnected network [32] (Figure 6). This is in good agreement with the perceived ability of the produced films to detach the plastic mold, in connection with the marked difference in the size and number of polygons outlined by the developed crack pattern [44]. Precursor films prepared without sorbitol (75-CRG/25-ALG) tended to spontaneously detach from the mold during oven drying, showing low adhesion to the plastic mold at this stage. Also, they appeared to be less prone to fracture during crosslinking and drying than the other produced materials, forming a small number of large (average size 205.7 ± 124.0 µm, Table 2) irregular polygons (Figure 6a). Conversely, these films appeared to exhibit stronger adhesion to the mold during crosslinking, which made complete detachment more difficult in some cases (e.g., Figure 3a). Increasing sorbitol concentration to 0.5% (Figure 6b) and to 1% (Figure 6c) resulted in polygons that are apparently larger in number and smaller in size (respectively, around 54 µm and 26 µm, Table 2). The same effect is obtained when 0.5% (75-CRG/25-ALG/50-SORB*, Figure 6d) and 1.0% (75-CRG/25-ALG/100-SORB*, Figure 6e) sorbitol is included in the respective crosslinking solutions. In this case, polygon size was around 39 µm and 14 µm, respectively, corresponding to an increasingly larger fracture surface. It should be mentioned that decreasing polygon size is accompanied by film thickness increase (around 43 µm, 46 µm and 51 µm for formulations with 0.50% and 100%, respectively) and linear retraction decrease from a maximum of 32% (no sorbitol) to 5.7 (50%) and 3.6% (100%) (Table 2). These features are expected to result from the viscosity increase brought about by the growing presence of sorbitol in the gel structure [40]. Because gel shrinkage is dominated by gel viscosity, higher stresses and higher strains act upon the films during drying, resulting in increased fracture on retraction and in thicker films [32].

**Figure 7:**
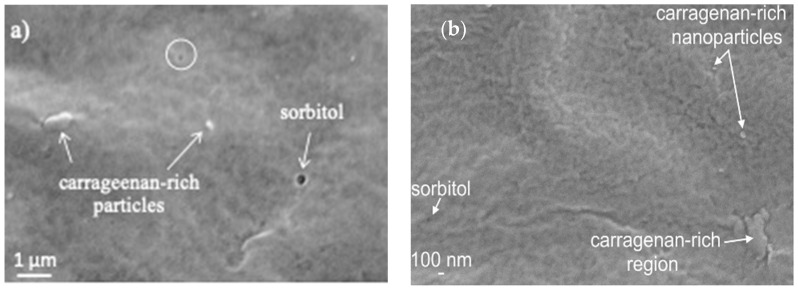
Microstructure of 75-CRG/25-ALG/50-SORB material: (**a**) film surface (circle highlights nanopores) and (**b**) high magnification image of the background, in the nanometric range (black: sorbitol; dark grey: alginate-rich phase; light grey: carrageenan-rich phase; white: carrageenan-rich particles).

Other than the effect of stress and strain developed during the drying of the gel films, which dominates surface morphology, sorbitol plasticizer also appears to have a strong influence on phase solubility and distribution. Comparison between the microstructure of 75-CRG/25-ALG (Figure 2b) and 75-CRG/25-ALG/50-SORB (Figure 6b) suggests higher miscibility between the two polysaccharides in the presence of sorbitol, apparently with lower numbers and smaller dimensions of carrageenan-rich particles. Figure 7a shows a high magnification image of the 75-CRG/25-ALG/50-SORB film surface: a uniform background is observed, displaying some scattered submicrometric particles and nanopores (Figure 7, circle). Figure 7b highlights the intimate mixture of alginate (dark grey), carrageenan (identified by Na/S ratio) and sorbitol (black, only C and O identified by EDS). The phase distributions appear to be very uniform, although some individualized carrageenan-rich particles (white) and sorbitol-rich domains (black) are visible.

The apparent increase of carrageenan/alginate miscibility, resulting in enhanced physical blending of the polymers, requires strong specific interactions [45]. This suggests that bonding established in the presence of sorbitol overcame the complex intramolecular cohesion of each polysaccharide and favored their miscibility [45]. The high number of carboxyl groups (alginate) and hydroxyl groups (carrageenan) enables hydrogen bonding and ionic interaction with sorbitol via its hydroxyl groups [19,38,45] establishing additional chemical bonds that are expected to stabilize intermolecular links [45].

**Figure 8:**
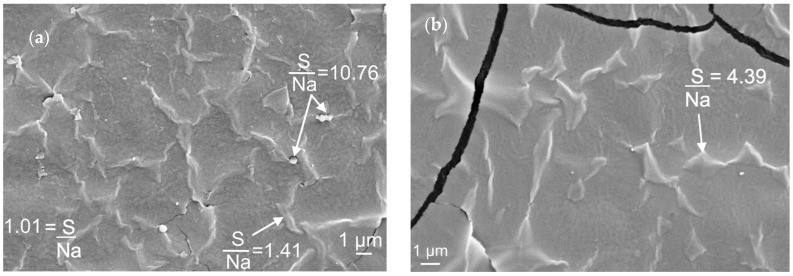
High magnification view of (**a**) 75-CRG/25-ALG/50-SORB* and (**b**) 75-CRG/25-ALG/50-SORB* film surface.

Yet, when sorbitol was included as a component of the crosslinking solution (films 75-CRG/25-ALG/50-SORB* and 75-CRG/25-ALG/100-SORB*, respectively; Figure 6d,e) the resulting microstructure was non-uniform, with formation of a cell-shaped [46], carrageenan-rich, secondary structure (Figure 8). The superstructure formed in films with lower sorbitol concentration (75-CRG/25-ALG/50-SORB*) appears to be more developed, with closed cell walls (Figure 8a). The background displays a S/Na weight ratio of around 1, the same as in 75-CRG/25-ALG films (Figure 2b), while it is higher (around 1.41) in the cell walls. Spherical particles with a diameter of around 200 nm and a very high S/Na weight ratio (10.76)—indicating a very high carrageenan fraction—are also visible (Figure 8a). In films richer in sorbitol (75-CRG/25-ALG/50-SORB*) the cell walls are apparently thicker, although somewhat open and incomplete (Figure 8b) and with a higher S/Na weight ratio (4.39) than before.

These results suggest that cell wall formation is an effect of phase separation in the gel, which is expected to take place when one of the gel components reaches a critical concentration [43,47]. At this point, repulsive interactions between chains trigger a macromolecular competition for space in the solution, leading to a volume exclusion effect and segregation [47]. The system reaches its thermodynamic minimum by phase separation, each one enriched in one of the polysaccharides [43]. This effect has often been reported regarding aqueous solutions of carrageenan/polysaccharide [43,47]. Yet, in this current work, no superstructures were detected in films with the same composition (75-CRG/25-ALG/50-SORB and 75-CRG/25-ALG/50-SORB) crosslinked without sorbitol (Figure 7). This signals that the formation of separated carrageenan superstructures is an outcome of the presence of sorbitol in the crosslinking solution. In this context, the supramolecular structure is expected to form when external sorbitol penetrates the gel and encounters uncoiled sections of carrageenan macromolecules, using its hydroxyl groups to creat intermolecular secondary bonds. Similar superstructures have been previously observed in κ-carrageenan/gelatin and sodium alginate/gelatin complexes [46].

#### 3.2.3. Compositional Analysis

**Figure 9:**
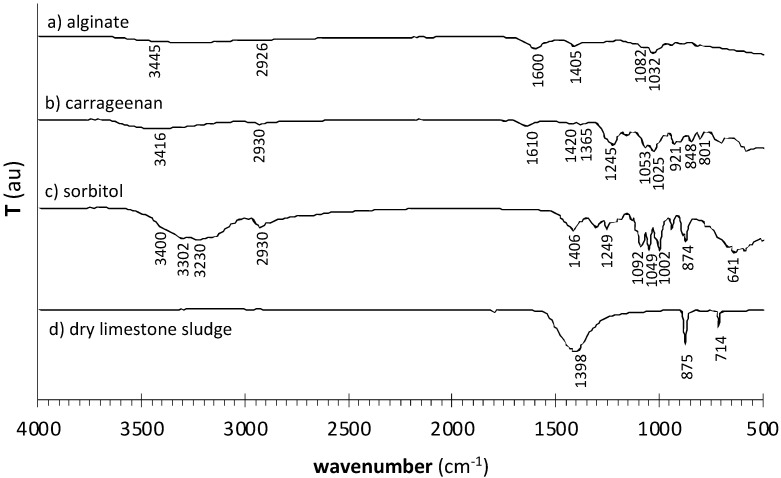
FTIR-ATR transmittance spectra of used reagents (in powder form): (**a**) sodium alginate, (**b**) ɩ-carrageenan and (**c**) D-(-)sorbitol. The spectrum of (**d**) dry limestone sludge used to prepare the crosslinking solutions is also shown.

Figure 9 displays the FTIR-ATR spectra of Na-alginate (Figure 9a), ɩ-carrageenan (Figure 9b) and D-(-)sorbitol (Figure 9c) reagents used in film preparation. As would be expected, some of the vibrations are common to the three polysaccharides: the broad band in the range 3350 ± 50 cm^−1^, assigned to the O–H stretch of the hydroxyl group [48]; the H–C–H asymmetric stretch of alkanes, with a maximum at 2926 ± 10 cm^−1^. The spectrum obtained for sodium alginate presents two strong bands that together confirm the presence of carboxylate groups at 1600 ± 50 cm^−1^ (O–C–O asymmetric stretch, more intense) and 1450 ± 50 cm^−1^ (symmetric stretch) [48,49]. The spectrum of sodium alginate (Figure 9a) further displays a significant absorption band around 1032 cm^−1^ [49], which may be attributed to C–O stretching vibrations. [48,49,50,51]. In the spectrum of ɩ-carrageenan (Figure 9b), the main diagnostic feature is the band around 801 cm^−1^, which indicates the presence of sulfate ester in the 2-position of the anhydro-D-galactose residues [52]. The band around 848 cm^−1^ was assigned to D-galactose-4-sulfate; the bands at ca. 1053 cm^−1^ and 921 cm^−1^ were assigned to the C–O stretch of the C3–O–C6 linkage of 3,6-anhydro-D-galactose [52]. The bands pertaining to the sulfate ester groups were also detected at 1241 cm^−1^ (stretch of S=O sulfate ester) [52]. Considering the available literature, the band observed at 1610 cm^−1^ was tentatively assigned to structural water deformation vibrations, which suggests the existence of moisture in the starting ɩ-carrageenan material [53,54,55]. The main vibration modes of the hydroxyl group in the spectrum of D-(-)sorbitol (Figure 9c) are the bands around 3230, 3302 and 3400 cm^−1^, corresponding to O–H stretching of the alcohol group (including free, inter- and intra-molecular bound hydroxyl groups [56]) and the out-of-plane O–H bending of the C–OH linkage, around 641 cm^−1^. Other significant bands display maximum absorption at 1406 cm^−1^ and 1249 cm^−1^ (respectively, the out-of-plane and in-plane bend of O–H of the C–OH linkage), 1082 and 1002 cm^−1^ (respectively, the asymmetric and symmetric stretch of C–O of the alcohol group) and 1049 cm^−1^ and 874 cm^−1^ (respectively, the asymmetric and symmetric C–C–O stretch of the alcohol group) [48,57]. The spectrum of the dry limestone sludge used to prepare the crosslinking solution is also shown (Figure 9d). All the bands are compatible with the vibration modes of the carbonate ion of CaCO_3_ (calcite polymorph): asymmetric stretching of about 1400 cm^−1^, out-of-plane bending of around 875 cm^−1^ and in-plane bending of around 700 cm^−1^ [58].

Expectedly, interactions established between reagents in subsequent crosslinking and drying mainly take place via hydrogen bonding of reactive hydroxyl groups in the polymer chains [16,38]. Any molecule that undergoes hydrogen bonding displays particularly broad and intense bands due to O–H stretch (3500–3200 cm^−1^) and O–H bend (650 ± 50 cm^−1^) [48], which are, in fact, the larger bands in the spectrum of D-(-)sorbitol (Figure 9c). This results from the highly variable number and strength of contributing hydrogen bonds, from place to place in alcohol molecules [48]. For this reason, the corresponding infrared bandwidths and intensities are much higher in the alcohol group in sorbitol than in other functional groups present in the raw materials (Table 3) [48]. Regarding the carboxylate groups of alginate, the spectrum of alginate also presents a broad and intense O–H stretching band [48], yet much less intense and somewhat shifted to the right.

**Figure 10:**
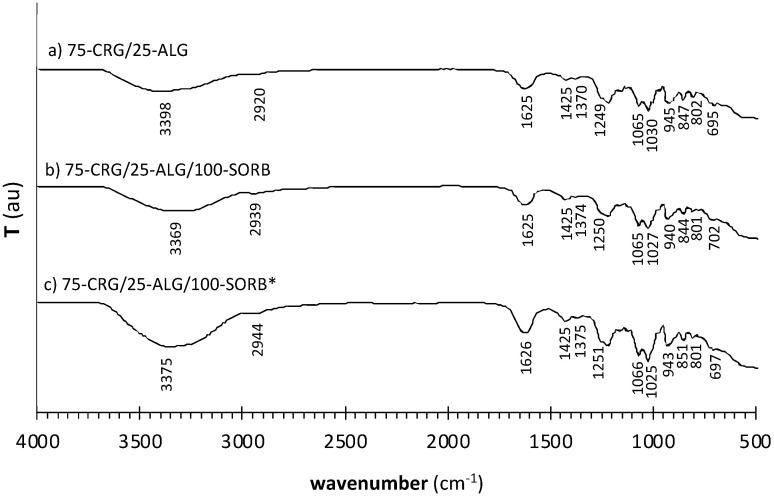
ATR-FTIR transmittance spectra of produced film materials: (**a**) 75-CRG/25-ALG, (**b**) 75-CRG/25-ALG/100-SORB (no sorbitol in the crosslinking solution) and (**c**) 75-CRG/25-ALG/100-SORB* (crosslinked in the presence of sorbitol).

After identification of the chemical structures giving rise to characteristic absorption bands in the reagents, the same procedure was followed for produced films, aiming to identify functional groups (Figure 10) involved in polysaccharide/polysaccharide and polysaccharide/plasticizer crosslinking.

All produced materials display a broad band in the 3350 ± 50 cm^−1^ range, associated with the O–H stretching vibration. Overall, band intensity and width increase with increasing sorbitol addition (Table 3) due to the increase of available hydroxyl groups. However, 75-CRG/25-ALG films (Figure 10a) do not contain sorbitol, while pure alginate (Figure 9a) and pure carrageenan (Figure 9b) are expected to contribute very little to band intensity and width, based on the low intensity of the O–H stretch band in pure reagents. In as much, the considerable size of the O–H band displayed by the 75-CRG/25-ALG material at 3382 cm^−1^ (Table 3) is expected to result from the disruption of intermolecular bonds between polymer chains and their substitution by water/alginate and water/carrageenan hydrogen bonds [38]. The frequency maximum of the O–H stretch band in the 75-CRG/25-ALG material shifts to a lower wavenumber than in pure reagents (Table 4), which is expected to correspond to a bond length increase [16]. This confirms the plasticizer effect of water, which leads the system to a “soften state” (as if above glass transition) that enables lower system viscosity and higher molecular mobility [13,16,37,38]. On its turn, the O–H stretch band broadens and increases in intensity (Table 3) when sorbitol is added both to 75-CRG/25-ALG/100-SORB and to 75-CRG/25-ALG/100-SORB* materials. Again, the increase of hydroxyl groups provided by the polyol and by the accompanying adsorbed water increases the number of hydrogen bonding in the films (Brian C Smith, 2018; Gao et al., 2017); the O–H stretch band maximum shifted to a lower wavenumber than in pure reagents (Table 4), corresponding to bond length increase [16].

The O–C–O stretch of alginate (around 1600 cm^−1^ and 1405 cm^−1^) shifted to a higher wavenumber when reagents were blended into the 75-CRG/25-ALG material (1625 cm^−1^ and 1450 cm^−1^) (Table 4). Bonding occurs via electrostatic interactions and hydrogen bridges, making bond length and intermolecular spacing decrease so that the spatial network structure becomes increasingly compact [19]. The introduction of sorbitol as a component of the film (75-CRG/25-ALG/100-SORB) and the inclusion of sorbitol as a component of the crosslinking solutions for the sorbitol-containing film (75-CRG/25-ALG/100-SORB*) plasticizer did not appear to interfere significantly with these bonds since the bands’ maxima position was approximately constant. The sulfate ester and cyclic ether groups in ɩ-carrageenan also appeared to be influenced by the metal-induced crosslinking: bands at around 1241 cm^−1^ and 1053 cm^−1^ shift, respectively, to 1249 cm^−1^ and 1065 cm^−1^ in the 75-CRG/25-ALG material (Table 4).

**Figure 11:**
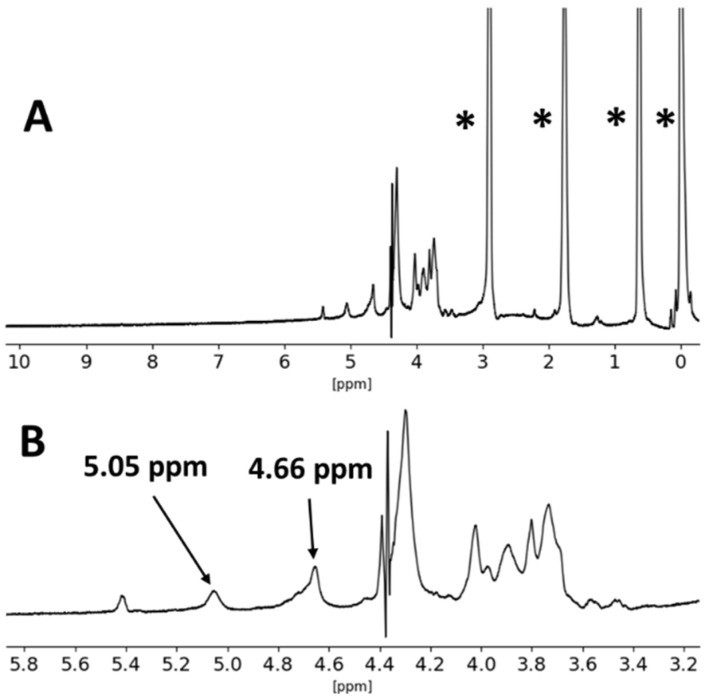
^1^H-NMR spectrum of the sodium alginate used as starting material, measured in D_2_O, at 60 °C (**A**), and magnification of the region between δ 5.8 ppm and 3.2 ppm (**B**). The asterisks (*) denote the DSS reference chemical shifts.

**Figure 12:**
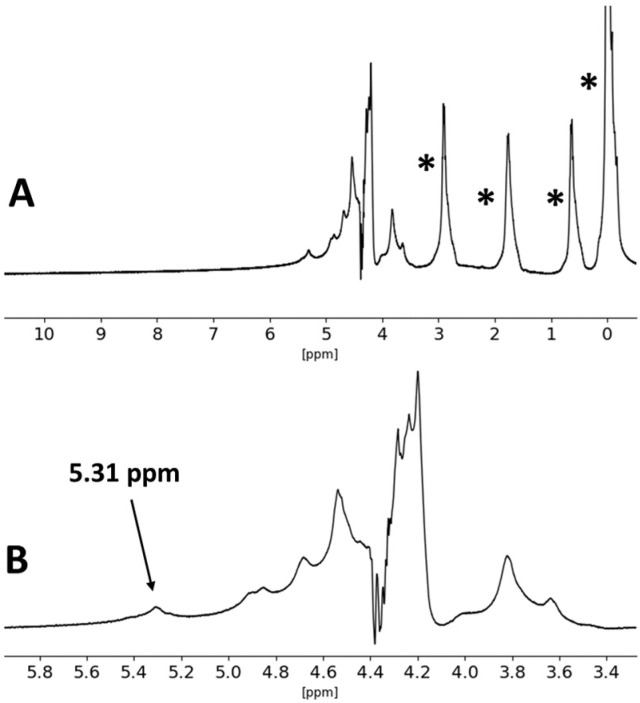
^1^H-NMR spectrum of the ɩ-carrageenan used as starting material, measured in D_2_O, at 60 °C (**A**), and magnification of the region between δ 5.8 ppm and 3.4 ppm (**B**). The asterisks (*) denote the DSS reference chemical shifts.

Analysis by NMR of the commercial alginate and ɩ-carrageenan was also carried out, and the obtained spectra are presented in Figure 11 and Figure 12.

The ^1^H-NMR spectra of commercial alginate presented the expected chemical shifts in the anomeric proton region between 5.4 ppm and 4.5 ppm. Namely, the shift observed at ca. d 5.05 ppm was assigned to the anomeric protons of the guluronic acid units (G), whereas the group of unresolved overlapping shifts at ca. d 4.7 ppm were tentatively assigned to the anomeric protons of the mannuronic acid units (M) and H5 protons of guluronic/mannuronic acid blocks [59,60]. However, the signal at ca. d 5.4 ppm could not be identified.

Due to generalized signal broadening and spectrum distortion in the d 4–4.5 ppm range, the M/G ratio of the analyzed alginate could not be estimated.

The obtained spectrum for ɩ-carrageenan is consistent with what is reported in the literature for this type of carrageenan. Taking into account the measurement temperature of 60 °C, the chemical shift observed at d 5.31 ppm was assigned to the anomeric protons of the iota-type carrageenan. No additional chemical shifts could be observed in the d 5–6 ppm range [61,62,63,64].

In light of the data obtained by NMR and ATR-FTIR, it was not deemed necessary to further purify the starting alginate and carrageenans prior to the preparation of the films.

#### 3.2.4. Films’ Mechanical Performance

**Figure 13:**
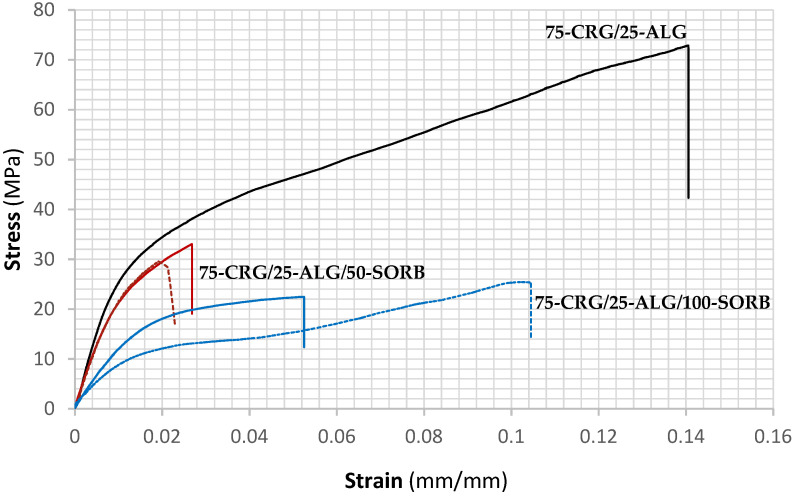
Representative tensile curves obtained for —75-CRG/25-ALG, —75-CRG/25-ALG/50-SORB and —75-CRG/25-ALG/100-SORB films. The corresponding films crosslinked in the presence of sorbitol are displayed in a dashed line: —75-CRG/25-ALG/50-SORB*, and —75-CRG/25-ALG/100-SORB*.

Suitable mechanical properties are an essential requirement of packaging materials. In as much, tailoring formulations for packaging applications requires knowledge of the effect of the selected plasticizer upon mechanical properties of the developed materials. The materials developed and produced in this current work present tensile curves with typical hyperplastic behavior and well-defined elastic/plastic transition. Figure 13 displays representative 75-CRG/25-ALG, 75-CRG/25-ALG/50-SORB* and 75-CRG/25-ALG/100-SORB* curves; similar behavior was found for the remaining films.

In good agreement with the typical brittleness and friability of produced films, fracture strain ranged from 1.8% to 14.7% (Table 5) and no correlation between the films’ behavior and added sorbitol concentration was found. In fact, Figure 13 shows a behavior opposite of the expected [65], with films’ fracture strain decreasing with increasing amount of sorbitol. The remaining mechanical properties (Table 5) were analyzed using the Bonferroni pairwise comparison tool, with statistically significant differences (*p* < 0.05) identified between different compositions. It should be mentioned that the delicate films were difficult to cut uniformly into specimens and hard to fix on the testing machine grips. This should explain the poor test reproducibility regarding mechanical properties.

**Figure 14:**
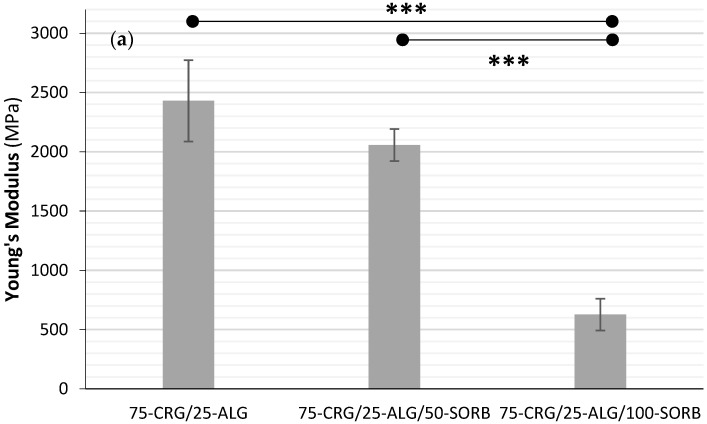

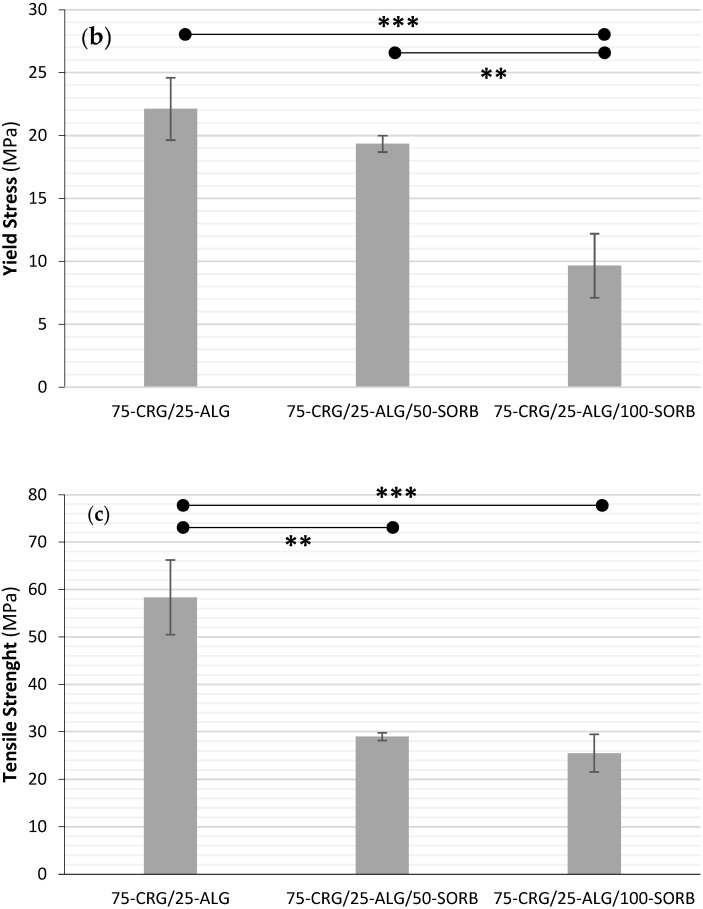
Influence of D-(-)sorbitol’s concentration on films’ mechanical performance: (**a**) Young’s modulus, (**b**) yield stress and (**c**) tensile strength (statistically significant difference: ** *p* < 0.01, *** *p* < 0.001).

The films’ Young’s modulus decreased from 2430 to 627 MPa as the concentration of D-(-)sorbitol increased (Figure 14a). Strong statistically significant difference (*p* < 0.001) was observed between 75-CRG/25-ALG, 75-CRG/25-ALG/50-SORB* and 75-CRG/25-ALG/100-SORB*. Yet, no statistically significant difference (*p* > 0.05) was found between the 75-CRG/25-ALG and 75-CRG/25-ALG/50-SORB* films, with high variation obtained for this property. Similar results were found for yield stress (Figure 14b), which decreased from 22.1 MPa to a minimum of 9.7 MPa with increasing sorbitol concentration. The results obtained for formulation 75-CRG/25-ALG/100-SORB are in line with reports in the literature regarding the influence of alginate and k-carrageenan on sodium caseinate-based films plasticized with glycerol: films containing alginate displayed a higher Young’s modulus (723–899 MPa) than k-carrageenan containing films (637–678 MPa) [66]. Finally, films without D-(-)sorbitol (75-CRG/25-ALG) displayed a maximum tensile strength of around 58 MPa (Figure 14c), which decreased to a minimum of 26 MPa (around 56%) when D-sorbitol was introduced (75-CRG/25-ALG/100-SORB film). A strong statistically significant difference was found between 75-CRG/25-ALG and 75-CRG/25-ALG/50-SORB (*p* = 0.00334) and between 75-CRG/25-ALG and 75-CRG/25-ALG/100-SORB (*p* = 0.00011)). Due to their low average value and high standard deviation, no statistically significant difference was found between 75-CRG/25-ALG/50-SORB and 75-CRG/25-ALG/100-SORB (*p* > 0.1). When correlated with the films’ thickness (Table 2), thinner films (0 and 50% sorbitol) displayed higher mechanical performance, possibly due to their tighter and enhanced intermolecular structure [67]. The attained film tensile strength values are in good agreement with the literature [68].

Overall, the use of plasticizer decreased Young’s modulus, yield stress and tensile strength values. This behavior is in line with previous work in porphyran-based films plasticized with glycerol or sorbitol [69] and in alginate and κ-carrageenan films using glycerol as a plasticizer [65].

**Figure 15:**
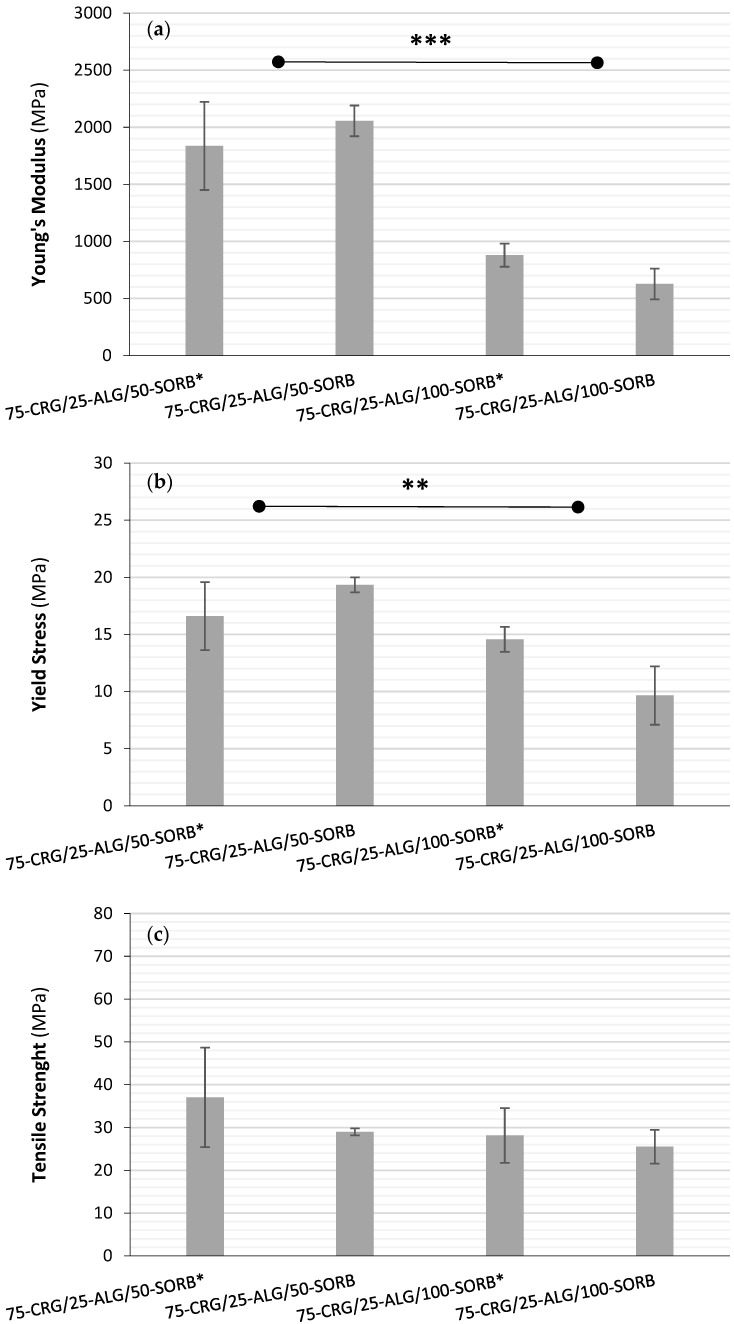
Influence of the presence of D-(-)sorbitol on the films’ crosslinking solution regarding (**a**) Young’s modulus, (**b**) yield stress and (**c**) tensile strength (statistically significant differences ** *p* < 0.01, *** *p* < 0.001). The asterisk (*) indicates that sorbitol was present in crosslinking solution.

The comparison of films with similar base composition crosslinked in the presence of sorbitol showed no statistically significant difference (*p* > 0.1) regarding the films’ Young’s modulus (Figure 15a), either between 75-CRG/25-ALG/50-SORB and 75-CRG/25-ALG/50-SORB*, or between 75-CRG/25-ALG/100-SORB and 75-CRG/25-ALG/100-SORB*. Still, the films with higher D-(-)sorbitol concentration (100%) showed increased flexibility (*p* < 0.00051). Similar results were obtained for the films’ yield stress (Figure 15b), although a two-fold increase in D-(-)sorbitol concentration only corresponded to an average 41% decrease in the yield stress (*p* < 0.00243). Finally, no statistically significant difference (*p* > 0.05) was found between the tensile strength of all the films (Figure 15c). Based on these results, it can be concluded that the presence of D-(-)sorbitol on the crosslinking solution does not significantly affect the film’s mechanical properties, probably due to the short crosslinking period and/or phase separation.

All produced materials showed friable, stiff and brittle behavior, although the maximum 14.7% fracture strain is an enhancement when compared to previous films obtained by the authors (3%) [70]. Brittleness is attributed to the highly fractured mud-crack surface displayed by all the films (Figure 6) [65], where long and slightly deflected cracks can be found independently of the presence of sorbitol. As mentioned, no correlation was found between the presence or absence of D-(-)sorbitol in the films and fracture strain values, yet SEM images indicate that increasing total sorbitol concentration in the films increases surface crack density (Figure 6).

#### 3.2.5. Anti-Fungal Properties

**Figure 16:**
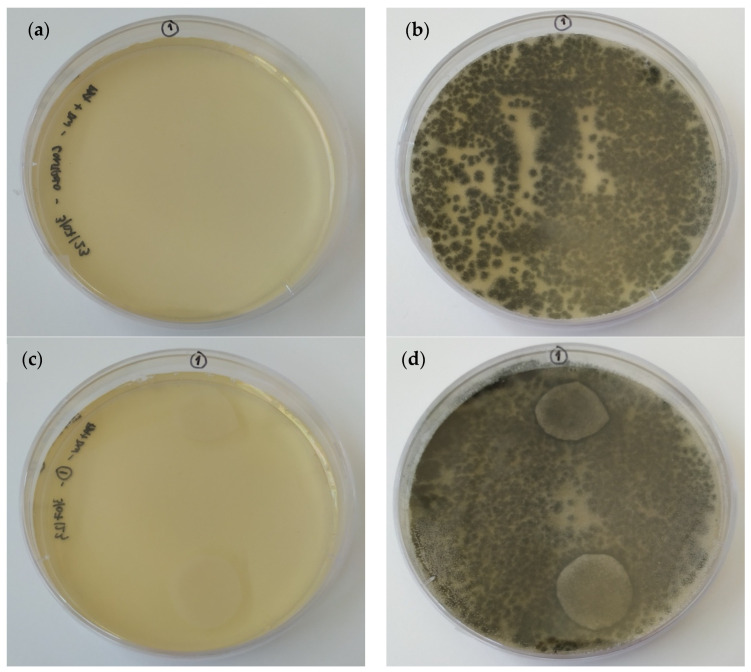
Inoculated plates without (**a**,**b**) and with (**c**,**d**) 75-CRG/25-AL film disks after 0 (**a**,**c**) and 7 days (**b**,**d**), at 28 °C.

The absence of inhibition halos around the film disks in the triplicates (Figure 16) suggests that the inclusion of ɩ-carrageenan in the crosslinked film did not have any inhibitory effect against *A. fumigatus* growth. The fact that some types of carrageenans have previously demonstrated antimicrobial properties [71,72] highlights the importance of considering the specific characteristics and properties of different carrageenan types, as they can vary in their effect on microbial growth.

## 4. Conclusions

Limestone sludge was successfully converted into a usable Ca^2+^ source for the crosslinking of alginate and ɩ-carrageenan/alginate films. As mentioned earlier, the content of calcium depends on the chemical composition of the starting sludge and the completeness of the conversion procedure. However, the full and in-depth chemical constitution of the starting sludge is necessary to assess the viability of its use, with regard to overall calcium content and the presence of toxic elements. Depending on the purpose of application of the film, if it is in the food area, the restrictions are greater and it will be necessary to carry out migration tests.

The crosslinked films based on alginate/sorbitol were dimensionally unstable after exposure to ambient conditions. The deformation was attributed to the formation of three-dimensional calcium–alginate gel structures which may lead to size contraction of the film structure. As such, the methods and formulations based on alginate/sorbitol were considered unsuitable for the preparation of films using the converted limestone crosslinking agent.

In contrast, the crosslinked films based on a 75/25 ɩ-carrageenan/alginate blend presented superior stability and overall better visual appearance compared to the alginate films. FTIR-ATR studies of these films revealed data consistent with ɩ-carrageenan/alginate and ɩ-carrageenan/alginate/sorbitol blends. The NMR analysis of the starting polysaccharides revealed data consistent with what is reported in the literature for sodium alginate and ɩ-carrageenan. The mechanical studies suggested that the inclusion of increasing amounts of D-(-)sorbitol as a plasticizer in the formulation resulted in a gradual lowering of the overall mechanical strength of the films. Further optimizations of the amount of plasticizer added are required to obtain an optimal trade-off between mechanical strength and flexibility.

No antifungal activity against *Aspergillus fumigatus* could be observed with the tested 75/25 ɩ-carrageenan/alginate blend films. Antimicrobial activity may be conferred by the incorporation of compounds with known antimicrobial activity, but the development of active films was outside of the scope of this work.

While the 75/25 ɩ-carrageenan/alginate blend films show preliminary potential for application as non-edible packaging, in-depth testing of aspects such as water vapor resistance and permeation, surface wettability and overall migration of components/contaminants should be performed. Nevertheless, the ɩ-carrageenan/alginate blend films described herein may serve as a starting point for the development of viable alternatives for film packaging based on conventional plastics, while simultaneously providing alternatives for the valorization of limestone byproducts.

## Figures and Tables

**Table 1:** Processing conditions and composition of the films prepared in this work.

Formulation	Crosslinking Solution	ALG/S_polysach_ (Weight Ratio)	CRG/S_polysach_ (Weight Ratio)	SORB/S_polysach_ (Weight Ratio)
100-ALG/100-SORB *	Previous hydration Ca^2+^ + sorbitol	100	0	100
	Ca^2+^ + sorbitol	100	0	100
75-CRG/25-ALG	Ca^2+^	75	25	0
75-CRG/25-ALG/50-SORB	Ca^2+^	75	25	50
75-CRG/25-ALG/100-SORB	Ca^2+^	75	25	100
75-CRG/25-ALG/50-SORB *	Ca^2+^ + sorbitol	75	25	50
75-CRG/25-ALG/100-SORB *	Ca^2+^ + sorbitol	75	25	100

S_polysach_: total algal polysaccharide mass; ALG: sodium alginate; CRG: ɩ-carrageenan; SORB: D-(-)sorbitol. The asterisk (*) indicates that sorbitol was present in crosslinking solution.

**Table 2:** Measured dimensional features of produced films.

Formulation	Film Thickness (µm)	Linear Retraction (Radial Direction) (%)	Polygon Average Size (µm)
75-CRG/25-ALG	44.7 ± 5.0	14.2 ± 4.9	205.7 ± 124.0
75-CRG/25-ALG/50-SORB	50.8 ± 7.6	8.3 ± 2.4	54.4 ± 22.0
75-CRG/25-ALG/100-SORB	52.1 ± 5.8	5.1 ± 2.5	38.8 ± 11.1
75-CRG/25-ALG/50-SORB *	48.1 ± 5.2	4.4 ± 1.9	25.6 ± 10.1
75-CRG/25-ALG/100-SORB *	55.0 ± 4.2	3.5 ± 2.1	14.4 ± 4.9

The asterisk (*) indicates that sorbitol was present in crosslinking solution.

**Table 3:** Measured band width and intensity corresponding to the O–H stretching mode (3500 ± 50 cm^−1^) in the used reagents and in produced films.

	Band Maximum Position (cm^−1^)	FWHM (cm^−1^)	T (%)
Na-alginate	3445	352 ± 6	7.6 ± 0.3
ɩ-carrageenan	3416	340 ± 1	9.4 ± 0.8
D-(-)sorbitol	3400	364 ± 3	34.0 ± 1.8
75-CRG/25-ALG	3398	415 ± 9	24.2 ± 0.9
75-CRG/25-ALG/100-SORB	3369	424 ± 5	22.6 ± 1.0
75-CRG/25-ALG/100-SORB *	3375	425 ± 3	46.8 ± 1.4

FWHM: full width half maximum; T: transmittance. The asterisk (*) indicates that sorbitol was present in crosslinking solution.

**Table 4:** Summary of FTIR-ATR results, displaying the more relevant vibration modes and band assignment of used reagents and produced films [41,48,49,52,53,54,55].

ALG	CRG	SORB	CRG/ALG	CRG/ALG/SORB	CRG/ALG/SORB*	Vibration Assignment
3445	3416	3400	3378	3369	3371	O–H stretch
2926	2930	2930	2920	2916	2914	C–H asymmetric stretch of alkanes
1600			1625	1626	1626	O–C–O asymmetric stretch of –COO^−^ (alginate)
	1610					Water deformation
1405			1450	1450	1450	O–C–O symmetric stretch of –COO^−^ (alginate)
		1406				Out-of-plane bend of O–H of C–OH linkage
	1245		1249	1250	1251	S=O stretch of ester sulfate
		1249				In-plane bend of O–H of C–OH linkage
		1082				Asymmetric stretch of C–O of alcohol
1082						C–O–C stretch
	1053		1065	1072	1066	C–O–C stretch of 3,6-anhydrogalactose
1032			1030	1027	1025	C–O stretch
	921		922	920	931	C–O stretch of 3,6-anhydrogalactose
	848		847	844	851	C–O-SO_3_ stretch of sulfate on C-4 of the galactose
	801					C–O-SO_3_ stetch of sulfate on C-2 of the 3,6-anhydrogalactose

CRG/ALG: crosslinked 75-CRG/25-ALG material; CRG/ALG/SORB: crosslinked 75-CRG/25-ALG/100-SORB; CRG/ALG/SORB*: crosslinked 75-CRG/25-ALG/100-SORB* (crosslinking solution containing sorbitol).

**Table 5:** Average value of films’ mechanical properties.

Film Designation	Young’s Modulus (MPa)	Yield Stress (MPa)	Tensile Strength (MPa)	Fracture Strain (%)
75-CRG/25-ALG	2430 ± 343	22.1 ± 2.5	58.3 ± 7.9	12.6 ± 3.0
75-CRG/25-ALG/50-SORB	2056 ± 135	19.3 ± 0.7	29.0 ± 0.8	1.8 ± 0.5
75-CRG/25-ALG/100-SORB	627 ± 134	9.7 ± 2.5	25.5 ± 3.9	12.7 ± 4.2
75-CRG/25-ALG/50-SORB *	1837 ± 386	16.6 ± 3.0	37.1 ± 11.6	9.2 ± 4.1
75-CRG/25-ALG/100-SORB *	879 ± 101	14.6 ± 1.1	28.2 ± 6.4	14.7 ± 2.1

The asterisk (*) indicates that sorbitol was present in crosslinking solution.

## Data Availability

Data are contained within the article.
